# An HLA-C amino-acid variant in addition to HLA-B*27 confers risk for ankylosing spondylitis in the Korean population

**DOI:** 10.1186/s13075-015-0855-3

**Published:** 2015-11-27

**Authors:** Kwangwoo Kim, So-Young Bang, Seunghun Lee, Hye-Soon Lee, Seung-Cheol Shim, Young Mo Kang, Chang-Hee Suh, Celi Sun, Swapan K. Nath, Sang-Cheol Bae, Tae-Hwan Kim

**Affiliations:** Department of Rheumatology, Hanyang University Hospital for Rheumatic Diseases, 222-1, Wangsimni-ro, Seongdong-gu, Seoul, 04763 Republic of Korea; Department of Radiology, Hanyang University Hospital for Rheumatic Diseases, 222-1, Wangsimni-ro, Seongdong-gu, Seoul, 04763 Republic of Korea; Division of Rheumatology, Daejeon Rheumatoid & Degenerative Arthritis Center, Chungnam National University Hospital, 282, Munhwa-ro, Jung-gu, Daejeon, 35015 Republic of Korea; Division of Rheumatology, Department of Internal Medicine, Kyungpook National University School of Medicine, 680, Gukchaebosang-ro, Jung-gu, Daegu, 41944 Republic of Korea; Department of Rheumatology, Ajou University School of Medicine, 164, World cup-ro, Yeongtong-gu, Suwon, 16499 Republic of Korea; Arthritis and Clinical Immunology Research Program, Oklahoma Medical Research Foundation, 825 NE 13th St., Oklahoma City, OK 73104 USA

## Abstract

**Introduction:**

The presence of the HLA-B*27 allele is a major risk factor for the development of ankylosing spondylitis (AS), which causes chronic inflammation of the spine and other sites. We investigated residual effects outside *HLA-B* within the major histocompatibility complex (MHC) region in the Korean population.

**Methods:**

Using the Korean HLA reference panel, we inferred the classic HLA alleles and amino-acid residues of the six HLA genes (*HLA-A*, *-B*, *-C,-DPB1*, *-DQB1*, and -*DRB1*) and MHC single-nucleotide polymorphisms in 3820 Korean subjects, including 654 Korean cases of AS and 3166 controls, who were genotyped by using Immunochip. Logistic regression and log-likelihood ratio tests were used in AS association tests for imputed markers.

**Results:**

The most significant associations were identified at amino-acid positions in the epitope-binding site of HLA-B (*P* = 1.71 × 10^−481^ at position 70, *P* = 7.20 × 10^−479^ at position 97, and *P* = 2.54 × 10^−484^ at positions 114), highlighting the risk effect of the HLA-B*27 allele and the protective effects of other classic alleles. A secondary effect was located at the leucine at amino-acid position 116 in the epitope-binding site of HLA-C (*P* = 1.69 × 10^−14^), completely tagging the HLA-C*15:02 allele. This residue had a large effect in HLA-B*27-negative patients (odds ratio = 6.6, 95 % confidence interval = 3.8 to 11.4).

**Conclusions:**

The four amino-acid positions of HLA-B and -C account for most of the associations between AS and MHC in the Korean population. This finding updates the list of AS susceptibility loci and provides new insight into AS pathogenesis mediated by MHC class I molecules.

**Electronic supplementary material:**

The online version of this article (doi:10.1186/s13075-015-0855-3) contains supplementary material, which is available to authorized users.

## Introduction

Ankylosing spondylitis (AS) is a highly heritable rheumatic disease causing chronic inflammation of axial spine, joints, and various organs. The presence of HLA-B*27 is strongly associated with the development of AS, although the underlying mechanism remains unclear [1]. The HLA-B*27 allele is found in approximately 90 % of patients [1], possibly explaining most of the genetic associations between AS and the major histocompatibility complex (MHC) locus on human chromosome 6.

Some evidence of multiple independent effects within the MHC locus had been suggested by association analysis using single-nucleotide polymorphisms (SNPs) or classic two- or four-digit alleles of human leukocyte antigen (HLA) genes [1–4]. Recently, our understanding of the AS-MHC association was further extended by Cortes et al., who reported a fine-mapping study assessing for the first time the AS associations of SNP, HLA amino-acid residues, and HLA classic alleles simultaneously in European populations [5]. The strongest association was observed for HLA-B amino-acid position 97 in the epitope-binding groove. There were six possible amino-acid residues at the position and their relative disease effects widely varied (Asn >> > Thr > Arg ≈ Tyr > Ser > Val), which explained the known association of HLA-B*27 and other HLA-B alleles based on the six amino-acid residues (HLA-B*27 > > HLA-B*51 > … > HLA-B*07 and HLA-B*08 > HLA-B*57) [5]. In addition, a novel association passing the genome-wide significance threshold was identified at an *HLA-A* allele after controlling for the effect of *HLA-B* susceptibility alleles [5].

Here, we conducted a similar fine-mapping study for the AS-MHC association by using both SNP and HLA variants (classic alleles and amino-acid residues) in Korean subjects to investigate the difference of secondary effects between the Asian and European populations and to identify novel functional variants conferring risk of AS in addition to HLA-B*27 in the Asian population.

## Methods

### Subjects and SNP data

In total, 3820 unrelated Korean subjects comprising 654 cases of AS and 3166 controls were analyzed in this study. The patients with AS satisfied the modified New York criteria for AS diagnosis [6]. The study was approved by the institutional review board of Hanyang University Hospital for Rheumatic Diseases in Seoul, Korea. Written informed consent was obtained from each subject.

The customized SNP array (Immunochip) was used to obtain high-density SNP data for the extended MHC region in the study subjects. The Immunochip data were previously described in our recent studies [2, 7]. By standard quality-control procedures, SNPs with a minor allele frequency of at least 0.01, call rates of at least 0.95, and *P* values in Hardy-Weinberg disequilibrium tests of at least 5 × 10^−7^ in unrelated individuals with SNP call rates of at least 0.90 were analyzed in this study. The study subjects were ethnically homogeneous and showed no evidence of systemic bias or potential population substructure.

### HLA imputation and association analysis

Using the SNP2HLA script [8] and the Korean HLA reference panel [9], we imputed the two- and four-digit classic alleles and amino-acid residues of *HLA-A*, *-B*, *-C,-DRB1*, *-DPB1*, and *-DQB1* as well as MHC SNPs (spanning 25–35 Mb on chromosome 6 which this study aimed to examine variants within). The imputation provided genotype information of each classic HLA allele, HLA amino-acid residue, and SNP as a binary variable [8]. AS associations of all markers with a minor allele frequency of at least 1 % and imputation quality (PLINK INFO) of at least 0.8 were assessed by using logistic regression, adding the top 10 principal components as covariates. EIGENSTRAT software was used to calculate the principal components from linkage disequilibrium-pruned Immunochip-wide genotyped data for non-AS-associated loci. The known AS loci have been defined in a large AS Immunochip study [2]. AS associations at HLA amino-acid positions (or multiple markers) were assessed by using log-likelihood ratio tests (LRTs) comparing fits between null and full logistic regression models. The null model included the 10 principal components only; the full model also included dosage of amino-acid residues at the tested amino-acid position, excluding the most frequent residue to avoid colinearity among the residues. Conditional analyses were performed by adding the identified markers as covariates in logistic regression. HLA-B amino-acid haplotypes were extracted from long-range haplotypes in the imputed data. Haplotype-specific odds ratios (ORs) of developing AS for each haplotype were calculated by using logistic regression adjusting for the 10 principal components.

## Results

### Imputing variants in MHC

To dissect the associations between various MHC variants and AS, we analyzed high-density SNP data from 654 Korean cases of AS and 3166 controls. We imputed 4914 polymorphic variations, including 151 classic alleles and 656 amino-acid variants in *HLA-A*, *-B*, *-C,-DRB1*, *-DPB1*, and *-DQB1* as well as 4107 MHC SNPs on the basis of our ethnicity-matched HLA reference panel, which has been well validated to generate reliable imputation results from Korean genotype data [9–11].

Consistent with previous AS association studies, a high frequency of imputed HLA-B*27 allele carriers was observed in the AS cases (88.1 %) but not in the controls (4.5 %). The imputation-based carrier frequency in controls was similar to the genotyping-based frequency in a separate set of Korean subjects (n = 413) in our previous study (5.1 %) [9]. We note that the Korean HLA reference panel was reported to yield concordance rates of 93.4 % for two-digit alleles and 89.3 % for four-digit alleles of *HLA-B* between imputed and actual data in East Asians [9]. Using our study subjects, we observed an imputation accuracy of 97.4 % for the imputed HLA-B*27 carriage in 583 AS cases who were conventionally genotyped for HLA-B*27 carriage as previously described [12].

### AS associations at HLA-B amino-acid positions 70, 97, and 114

**Fig. 1:**
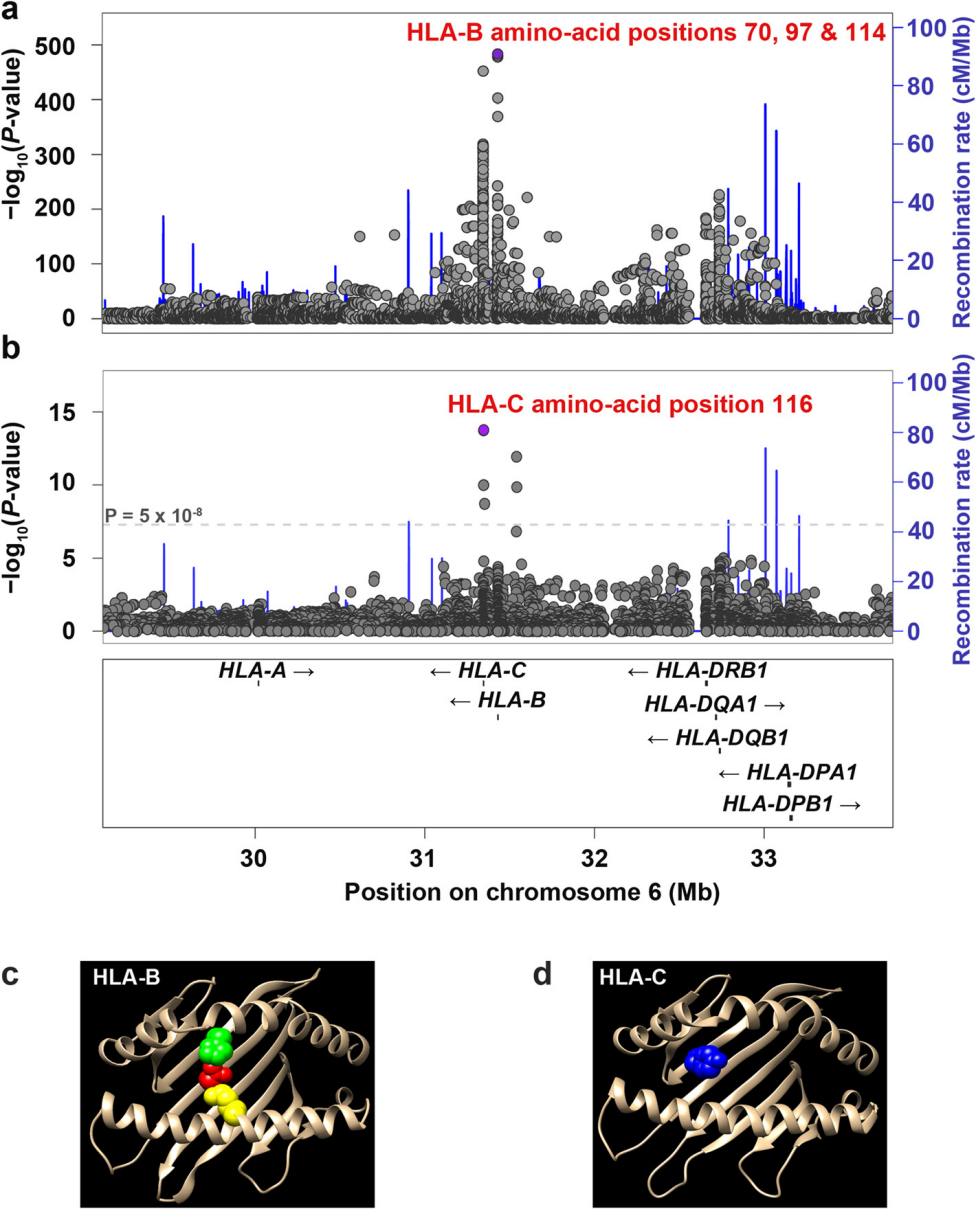
Association plots for the extended major histocompatibility complex region. Significance levels of each marker (single-nucleotide polymorphisms, classic HLA alleles, and HLA amino-acid residues) and amino-acid positions were calculated by using logistic regression and log-likelihood tests, respectively, and plotted according to chromosomal locations (based on hg18). **a** Primary association was identified at HLA-B amino-acid positions 70, 97, and 114 in an unconditional analysis. **b** Secondary association was identified at HLA-C amino-acid position 116 when conditioned on the primary effect at the three HLA-B amino-acid positions. **c, d** Amino-acid positions 70 (*yellow*), 97 (*red*), and 114 (*green*) of HLA-B and 116 of HLA-C (*blue*) are located in epitope-binding sites. *HLA* human leukocyte antigen

By simultaneously analyzing the associations for the three types of variant (SNPs, HLA amino-acid residues, and classic HLA alleles), we identified the most significant associations to be at HLA-B amino-acid positions 70, 97, and 114, and significance levels were highly reliable (*P*_LRT_ = 1.71 × 10^−481^ at 70, *P*_LRT_ = 7.20 × 10^−479^ at 97, and *P*_LRT_ = 2.54 × 10^−484^ at 114) and in fact stronger than those of any SNPs or classic HLA alleles, including HLA-B*27 (*P* = 2.11 × 10^−243^; Table 1 and Fig. 1a).

**Table 1:** Association of HLA-B amino-acid positions 70, 97, and 114 with susceptibility to ankylosing spondylitis

HLA-B association models	Null*	1	2	3	4	5	6	7
Amino-acid positions	-	70	97	114	70 + 97	97 + 114	70 + 114	70 + 97 + 114
AIC values	3503.0	1285.6	1282.7	1276.2	1282.5	1270	1280.1	1271.6
Log-likelihood test (LRT)								
*P* values in LRT (Ref = Null)	Ref	1.71 × 10^−481^	7.20 × 10^−479^	2.54 × 10^−484^	1.91 × 10^−477^	6.48 × 10^−480^	2.62 × 10^−480^	1.12 × 10^−478^
*P* values in LRT (Ref = model 1)	NA	Ref	NA	NA	0.023	NA	8.75 × 10^−3^	2.25 × 10^−4^
*P* values in LRT (Ref = model 2)	NA	NA	Ref	NA	0.10	2.44 × 10^−4^	NA	7.91 × 10^−4^
*P* values in LRT (Ref = model 3)	NA	NA	NA	Ref	NA	6.35 × 10^−3^	0.55	8.29 × 10^−3^
*P* values in LRT (Ref = model 4)	NA	NA	NA	NA	Ref	NA	NA	5.92 × 10^−4^
*P* values in LRT (Ref = model 5)	NA	NA	NA	NA	NA	Ref	NA	0.22
*P* values in LRT (Ref = model 6)	NA	NA	NA	NA	NA	NA	Ref	2.42 × 10^−3^
*P* values for conditioned amino-acid effects								
adjusted for position 70	NA	NA	0.1028	0.5455	NA	0.2195	NA	NA
adjusted for position 97	NA	0.023	NA	6.35 × 10^−3^	NA	NA	2.42 × 10^−3^	NA
adjusted for position 114	NA	8.75 × 10^−3^	2.44 × 10^−4^	NA	5.92 × 10^−4^	NA	NA	NA

*HLA* human leukocyte antigen, *AIC* Akaike information criterion, *Ref* reference model, *NA* not available

* The null model included the 10 principal components only

We then attempted to statistically distinguish which position is driving AS among the three positions (1) by conditioning the dosage of all residues, excluding the most frequent allele, at each position for the others and (2) by measuring the Akaike information criterion (AIC) and log-likelihood ratio among logistic models, including single, two, or three amino-acid positions. We observed dramatic drops in all *P* values for various combinations of amino-acid positions in the cross-conditional analysis adjusting for each of the three amino-acid positions (Table 1). In addition, we found similar AIC and *P*_LRT_ values for possible amino-acid models (Table 1) so that we could not statistically pinpoint certain positions among the three positions. Instead, these results strongly suggested that the haplotypes defined from the three positions could be highly stable. The three amino-acid positions are located in the epitope-binding pocket of HLA-B (Fig. 1c).

Considering strong linkage disequilibrium in HLA and uncertainty in imputation, we built an AS and HLA-B association model by using positions 70, 97, and 114 in a subsequent analysis. HLA-B amino-acid positions 70, 97, and 114 can be occupied by four, five, and three common amino-acid residues, respectively, and each residue had a distinct effect size (Additional file 1: Table S1). In total, 11 common amino-acid haplotypes (>1 % among the subjects) were defined by the three positions in this Korean study population. Haplotype association analysis showed that a single haplotype including lysine at 70, asparagine at 97, and histidine at 114 was significantly associated with increased susceptibility to AS (OR = 243.0; *P* = 2.70 × 10^−216^; Table 2), corresponding to HLA-B*27 alleles. Two HLA-B*27 alleles (HLA-B*27:04 and HLA-B*27:05) were observed in the Korean subjects in this study. In addition, the haplotype model explains how other haplotypes confer protection against AS. For example, the haplotype including asparagine at 70, tryptophan at 97, and asparagine at 114 indicated as HLA-B*14:01 had a highly protective effect (OR = 0.27; *P* = 4.34 × 10^−3^; Table 2).

**Table 2:** Association of HLA-B amino-acid haplotype with susceptibility to ankylosing spondylitis

Amino-acid haplotype			Haplotype frequency		Association for AS	
70	97	114	Cases	Controls	OR (95 % CI)	*P* value
Lys	Asn	His	0.482	0.025	243.0 (172.4–342.4)	2.70 × 10^−216^
Asn	Ser	Asn	0.064	0.087	0.72 (0.56–0.91)	6.38 × 10^−3^
Gln	Ser	Asp	0.024	0.038	0.65 (0.45–0.95)	2.55 × 10^−2^
Asn	Arg	Asn	0.064	0.103	0.59 (0.47–0.75)	1.49 × 10^−5^
Gln	Thr	Asn	0.042	0.069	0.58 (0.44–0.78)	2.77 × 10^−4^
Gln	Arg	Asp	0.024	0.044	0.54 (0.37–0.79)	1.24 × 10^−3^
Asn	Thr	Asn	0.121	0.217	0.48 (0.40–0.58)	2.02 × 10^−15^
Ser	Arg	Asp	0.031	0.065	0.46 (0.33–0.64)	3.70 × 10^−6^
Asn	Arg	Asp	0.141	0.319	0.35 (0.30–0.42)	2.74 × 10^−34^
Asn	Trp	Asn	0.004	0.014	0.27 (0.11–0.66)	4.34 × 10^−3^
Asn	Ser	Asp	0.001	0.012	0.06 (0.01–0.44)	5.69 × 10^−3^

*HLA* human leukocyte antigen, *AS* ankylosing spondylitis, *OR* odds ratio, *CI* confidence interval

*Each of the HLA-B classic alleles observed in the Korean subjects belongs to one of the amino-acid haplotypes. Lys-Asn-His: *27:04, *27:05; Asn-Ser-Asn: *08:01, *40:02, *48:01; Gln-Ser-Asp: *07:02; Asn-Arg-Asn: *13:01, *37:01, *38:02, *39:01, *40:01, *51:06; Gln-Thr-Asn: *54:01, *55:01, *55:02, *56:01, *56:05; Gln-Arg-Asp: *46:01; Asn-Thr-Asn: *13:02, *40:06, *51:01, *51:02, *52:01, *59:01; Ser-Arg-Asp: *58:01; Asn-Arg-Asp: *15:01, *15:02, *15:11, *15:18, *15:27, *15:38, *18:01, *35:01, *35:03, *44:02, *44:03; Asn-Trp-Asn: *14:01; Asn-Ser-Asp: *15:07, *40:03

We note that the AS association of HLA-B in European populations was explained by HLA-B amino-acid position 97 alone [5]. As compared with the most frequent residue, arginine, the effect size of asparagine residue (corresponding to HLA-B*27) was larger in the Korean population (OR = 38.8, 95 % CI = 31.5–47.7) than the European population (OR = 16.5, 95 % CI = 15.4–17.7) and serine showed opposite effects between the populations (OR = 1.28 in Koreans and OR = 0.86 in Europeans; *P*_Cochrane-Q test_ = 6.93 × 10^−4^) (Additional file 1: Table S2). It might mean that serine has a different effect on AS in different populations. Alternatively, the observed genetic heterogeneity of the serine residue between the Korean and European ancestries can be shown because of the presence of independent effects at HLA-B amino-acid position 70 or 114 or may indicate different secondary effects within the MHC region between the populations that can influence residue effects at position 97.

### AS associations at HLA-C amino-acid position 116

To identify independent genetic effects in addition to the *HLA-B* effects, we used a conditional logistic regression adding the three amino-acid positions 70, 97, and 114 (that is, the dosage of all residues at each position, excluding the most frequent allele) as covariates to remove their primary association effects. A secondary independent effect was identified at the leucine residue at HLA-C amino-acid position 116 located in the epitope-binding groove of HLA-C (*P* = 1.69 × 10^−14^; Fig. 1b and d). We note that the leucine residue was still most strongly associated with AS when adjusted for all four-digit *HLA-B* alleles (*P* = 1.21 × 10^−12^).

This residue was almost completely correlated with HLA-C*15:02 (dosage correlation *r*^2^ = 1.00, conditioned association *P* = 1.79 × 10^−14^) and enriched in AS patients without the HLA-B*27 allele (24.4 %), in contrast to AS patients carrying the allele (2.6 %) and the control group (4.3 %). As expected, the residue increased AS susceptibility in comparison with controls and HLA-B*27-negative cases (OR = 6.58, 95 % CI = 3.80–11.37; *P* = 1.52 × 10^−11^). This raises the possibility that another MHC class I molecule, HLA-C, has a complementary role in AS pathogenesis when the pathogenic HLA-B*27 molecules are absent.

We combined effects at the four amino-acid positions in HLA-B and -C into a single disease model. AS susceptibility was well explained by this model (*P*_LRT_ = 1.54 × 10^−487^), indeed significantly better than by the HLA-B amino-acid model (*P*_LRT_ = 1.12 × 10^−478^).

### No evidence of associations at *HLA-A* in Koreans

After conditioning on the four AS-associated amino-acid positions, we could not find any HLA variants associated with AS susceptibility (*P* > 1.19 × 10^−5^), passing the genome-wide significance threshold (*P* = 5 × 10^−8^). Outside of HLA genes, one MHC SNP rs11757571 on three prime untranslated region (3′-UTR) of *HCP5* showed a significant association (OR = 6.52; *P* = 2.78 × 10^−8^; INFO value = 0.99) but this association needs to be validated in an independent study because the significance was oddly depending on conditioning for HLA-B amino-acid position 70 among the three correlated HLA-B positions 70, 97, and 114 (after excluding HLA-B amino-acid position 70 from the covariates, OR = 2.42 and *P* for rs11757571 = 3.39 × 10^−5^).

Cortes et al. [5] have demonstrated a secondary association effect, exceeding the genome-wide significance threshold, at three correlated *HLA-A* markers (e.g., HLA-A*02:01; OR = 1.25 and *P* = 1.41 × 10^−9^) after controlling for the association of *HLA-B* alleles. This association was not detected in the Korean population but a similar conditioned effect was observed after adjusting for HLA-B*27 (OR = 1.25; *P* = 0.08), indicating possible sharing of risk alleles between European and Koreans. Alternatively, it is also possible that the sources of secondary effects could differ between Asian and Europeans. In our analysis, we found an HLA-C amino-acid variant with a second effect, but the unconditioned effect of HLA-A*02:01 (OR = 2.63; *P* = 2.12 × 10^−39^) was diminished after conditioning on *HLA-B* alleles (OR = 1.10; *P* = 0.47) or conditioning on HLA-B amino-acid positions 70, 97, and 114 (OR = 1.12; *P* = 0.43).

## Discussion

Adding HLA amino-acid variants and classic alleles in a fine-mapping study is very important for better localization of association effects to precise variants because the identification of primary and secondary effects highly depends on adjusting factors used in a conditional analysis. Our results illustrate the usefulness of adding HLA variants to an MHC fine-mapping analysis. Consistent with other studies that have investigated the trait associations down to the amino-acid level since early 2010 [13, 14], the independent disease-risk effects were identified at HLA amino-acid positions, providing a parsimonious and biologically relevant model.

We used the HLA imputation method and the Korean HLA reference panel with several types of genetic variant, including SNPs, two-digit HLA alleles, four-digit HLA alleles, and HLA amino-acid residues, to localize AS associations in the extended MHC region. The large effects were mapped primarily to HLA-B amino-acid positions 70, 97, and 114. They seemed partially linked with each other, and the three positions better explained the association of the established AS-risk HLA-B*27 allele and other protective alleles.

In addition, our findings revealed a novel pathogenic effect of HLA-C molecules, which can be exerted by leucine at amino-acid position 116 in the middle of the epitope-binding site. This residue was correlated with one of the HLA-C*15 alleles that have been suggested to be associated with Kawasaki disease, Behçet’s disease, and leprosy [15–17]. However, because this study had no replication cohort and previous European studies have not reported the association for HLA-C amino-acid position 116, this association needs to be validated in an independent cohort in the future.

We also demonstrate the difference of effect estimates at HLA-B amino-acid position 97 between populations and a possible difference of secondary association effects between Asian and European populations. Although both Korean and European populations have an independent disease effect at MHC class I molecules in addition to *HLA-B*, the association was mapped at *HLA-A* in Europeans but *HLA-C* in Koreans [5]. The relatively small size of the Korean subjects may cause the absence of the *HLA-A* association because of low statistical power to detect the effect size (OR = 1.25) observed in the European cohort (power = 80 % at the significance level of 0.05 or approximately 0 % at the significance level of 5 × 10^−8^ with the fixed allele frequency of 0.184 and the fixed disease prevalence of 0.5 %). Trans-ancestral association mapping could be useful in obtaining a robust profile of heterogenic associations resulting from ancestral differences.

## Conclusions

In summary, we identified associations of *HLA-C* in addition to *HLA-B* with AS susceptibility in the Korean population. This updates the list of AS susceptibility loci and provides new insight into AS pathogenesis mediated by MHC class I molecules.

## Additional file

Additional file 1: Table S1. Effect estimates for each residue at HLA-B amino-acid positions 70, 97, and 114. **Table S2.** Association of HLA-B amino-acid position 97 with ankylosing spondylitis in Korean and European populations. (PDF 84 kb)

## Abbreviations

AIC: Akaike information criterion
AS: Ankylosing spondylitis
CI: Confidence interval
HLA: Human leukocyte antigen
LRT: Log-likelihood ratio test
MHC: Major histocompatibility complex
OR: Odds ratio
SNP: Single-nucleotide polymorphism
