# Supplementary material for: An HLA-C amino-acid variant in addition to HLA-B*27 confers risk for ankylosing spondylitis in the Korean population
Source: Arthritis Res Ther. 2015 Nov 27;17:342. doi: 10.1186/s13075-015-0855-3 (PMC4662802; doi:10.1186/s13075-015-0855-3)
Supplement: Additional file 1: Table S1. — Effect estimates for each residue at HLA-B amino-acid positions 70, 97, and 114. Table S2. Association of HLA-B amino-acid position 97 with ankylosing spondylitis in Korean and European populations. (PDF 84 kb) [file 13075_2015_855_MOESM1_ESM.pdf]

Table S1. Effect estimates for each residue at HLA-B amino-acid positions 70, 97 and 114

Amino-acid position	Residue	Frequency in cases*	Frequency in controls*	OR	95% CI	*P*
70	Lys	0.465	0.026	294.16	206.89−418.23	6.08×10^{-220}
70	Gln	0.095	0.161	0.52	0.42−0.64	5.73×10^{-10}
70	Ser	0.034	0.068	0.46	0.33−0.64	3.22×10^{-06}
70	Asn	0.406	0.743	0.18	0.16−0.21	7.02×10^{-106}
97	Asn	0.465	0.027	291.35	205.13−413.79	1.56×10^{-220}
97	Ser	0.091	0.143	0.59	0.48−0.72	4.95×10^{-07}
97	Thr	0.164	0.286	0.47	0.40−0.56	1.10×10^{-19}
97	Arg	0.274	0.532	0.30	0.26−0.34	1.11×10^{-61}
97	Trp	0.006	0.011	0.26	0.08−0.84	0.024
97	Val	0.003	0.003	NA	NA	NA
114	His	0.465	0.026	293.73	206.70−417.39	1.64×10^{-220}
114	Asn	0.301	0.488	0.42	0.37−0.48	1.33×10^{-35}
114	Asp	0.234	0.486	0.29	0.25−0.34	5.07×10^{-59}

*Frequency was calculated from dosage data (but not from the most likely genotypes).
OR, odds ratio; CI, confidence interval; NA, not available.

Table S2. Association of HLA-B amino-acid position 97 with ankylosing spondylitis in Korean and European populations

	Frequency in Koreans*		Each vs the others		Each vs the reference (Arg)†		
Residue	Cases	Controls	OR (95% CI)	*P*	OR (95% CI)	*P*	OR (95% CI) in European‡
Asn	0.465	0.027	291.3 (205.1-413.8)	1.56×10^{-220}	351.7 (237.1-521.6)	8.03×10^{-187}	16.51 (15.43-17.69)
Ser	0.091	0.143	0.59 (0.48-0.72)	0.029	1.66 (1.13-2.44)	0.029	0.86 (0.81-0.91)
Thr	0.164	0.286	0.47 (0.40-0.56)	1.10×10^{-19}	1.27 (0.93-1.72)	0.130	1.12 (1.03-1.21)
Arg	0.274	0.532	0.30 (0.26-0.34)	1.11×10^{-61}	1 (reference)	NA	1 (reference)
Trp	0.006	0.011	0.26 (0.08-0.84)	0.024	0.15 (0.02-1.14)	0.067	1.00 (0.89-1.12)
Val	0.003	0.003	NA	NA	NA	NA	0.68 (0.59-0.78)

*Frequency was calculated from dosage data (but not from the most likely genotypes).
†The estimates were calculated using multivariate logistic regression.
‡European data were obtained from Cortes A et al (*Nature communications* 2015, 6:7146).
OR, odds ratio; CI, confidence interval; NA, not available.
